# Strategies for Reducing Salt and Sugar Intakes in Individuals at Increased Cardiometabolic Risk

**DOI:** 10.3390/nu13010279

**Published:** 2021-01-19

**Authors:** Valentina Ponzo, Marianna Pellegrini, Paola Costelli, Laura Vázquez-Araújo, Lucía Gayoso, Chiara D’Eusebio, Ezio Ghigo, Simona Bo

**Affiliations:** 1 Department of Medical Sciences, University of Turin, 10126 Turin, Italy; valeponzo1@yahoo.it (V.P.); mariannapellegrini87@gmail.com (M.P.); chiara.deusebio@edu.unito.it (C.D.); ezio.ghigo@unito.it (E.G.); 2 Department of Clinical and Biological Sciences, University of Turin, 10126 Turin, Italy; paola.costelli@unito.com; 3 BCC Innovation, Technology Center in Gastronomy, Basque Culinary Center, 20009 Donostia-San Sebastián, Spain; lvazquez@bculinary.com (L.V.-A.); lgayoso@bculinary.com (L.G.); 4 Basque Culinary Center, Faculty of Gastronomy Sciences, Mondragon Unibertsitatea, 20009 Donostia-San Sebastián, Spain

**Keywords:** cardiometabolic risk, diet strategies, salt reduction, sugar reduction

## Abstract

Non-communicable diseases (NCDs) are the first causes of death worldwide. Reduction in the dietary intake of salt and sugars is important lifestyle advice that is useful for NCD prevention. However, the simple recommendations of reducing salt and sugars by healthcare professionals are often ineffective; innovative strategies are therefore necessary. This review aimed at describing the current knowledge about the strategies to reduce dietary salt and sugar intake, including both strategies for the food industry to reduce the salt or sugar of its products and recommendations for health professionals in a clinical context, such as the replacement with substitutes in foods, the gradual reduction to allow a progressive consumer adaptation towards less intense taste, and the different spatial distribution of tastants within the food matrix with taste intensity enhancement. In addition, the cross-modal interaction between two or more different sensory modalities as an innovative strategy for enhancing sweetness and saltiness perception was described. Finally, the dietary tips for salt and sugar reduction were summarized in order to create a comprehensive guide of dietary advices for healthcare professionals for optimizing the management of patients at increased cardiometabolic risk.

## 1. Introduction

According to the World Health Organization (WHO) estimates, non-communicable diseases (NCDs) including cardiovascular disease, obesity, and diabetes mellitus, are responsible for almost 70% of all deaths and their prevalence is increasing worldwide [1]. Modifiable risk factors, such as unhealthy diets and physical inactivity, are among the causes of the rapid rise of NCDs prevalence [2]. Dietary recommendations for weight management and the prevention of chronic diseases include the proper intake of fruit and vegetables and the reduction of the intake of added sugars, unhealthy fats, salt, processed foods, and sugary drinks [3]. However, the compliance with these recommendations is frequently poor [4].

Changing eating behavior is a complex process. Simply advising the patients to reduce their intake of sugar and salt or to avoid certain foods does not work [5]. Many barriers hinder changes in dietary habits; among the most common are apathy and dietary conservatism, negative emotions and stress, lack of knowledge or support, false beliefs, confusing and misleading information provided by the media, difficulties in changing ways of cooking and in translating healthy eating messages into balanced food choice, cost of food, fear of waste, lack of confidence in cooking skills, personal taste, cultural acceptability, concerns relative to ease, speed of preparation, family acceptability, social relationships and approval [6,7,8,9]. Many of these factors can play a particularly relevant role in socially marginalized population groups with fewer resources to be able to face greater challenges and implement effective and lasting changes [8,9]. In the primary care setting, only a few physicians provided counselling on lifestyle modification, due to time constraints, the lack of specific nutrition education in medical school/residency training, and distrust in the patients’ willingness or ability to modify their diet [10]. In order to increase health-related attitudes on food choice and consequent nutrient intake, good communication is essential, and it is essential to target messages carefully and individually, by keeping information simple, relevant, realistic, meaningful, and practical for the specific person, by taking into account his/her attitudes, knowledge, cultural background, and behavior [6]. Furthermore, consistent efforts to correct misunderstandings as well as to lower the perceived barriers to dietary changes, and to improve self-confidence are needed. In particular, it is necessary to provide alternative and effective strategies for reducing the consumption of sugar and salt. A favorable environment allowing people to adopt healthy behaviors is a priority. Several population strategies have been experimented, such as the regulation of food labeling, a color code based on the salt content, different nutrition educational campaigns, and taxation policies (“sugar tax”) [11,12,13]. These strategies implied a government role and the involvement of many stakeholders.

**Figure 1:**
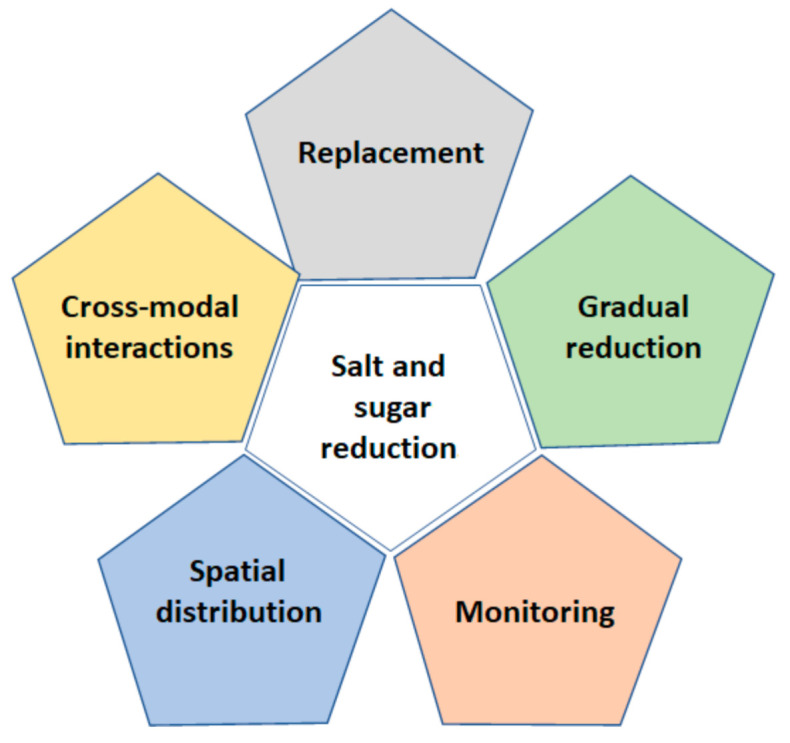
Strategies for salt and sugar reduction in foods.

In this review, we focused on the strategies that healthcare professionals can adopt in their daily practice in order to reduce the sugar and salt intake of their patients at increased risk for cardiometabolic diseases (Figure 1). In particular, the following topics were covered:

- How to reduce the dietary intake of salt
  - Dietary sources of salt intake;
  - Hidden sources of salt;
  - Replacing salt strategy;
  - Reduction strategy (gradual adaptation);
  - Spatial distribution strategy;
  - Cross modal interactions;
  - Dietary tips for salt reduction.
- How to reduce the dietary intakes of sugar
  - Dietary sources of sugar intake;
  - Hidden sources of sugar;
  - Replacing sugar strategy;
  - Spatial distribution strategy;
  - Cross modal interactions;
  - Dietary tips for sugar reduction.
- Future perspectives.
- Conclusive remarks.

## 2. Materials and Methods 

The following databases were queried: PubMed (National Library of Medicine), the Cochrane Library, and Excerpta Medica dataBASE (EMBASE). The search strategy was performed by using database-specific subject headings and keywords (i.e., sugar intake, sugar reduction, sugar strategies, salt intake, salt reduction, salt strategies, dietary strategies, dietary guidelines). Since this was a narrative review, the objectives were broad, and defined criteria for the selection or exclusion of studies were not applied; this represents a limitation common to all narrative reviews. Hand-searching the references of the studies and reviews of the field was performed to augment the search strategy. 

## 3. How to Reduce the Dietary Intake of Salt

### 3.1. Dietary Sources of Salt Intake

Excess salt intake is a major contributor to high blood pressure [14]. Salt reduction has been identified as one of the top five priority interventions to prevent NCDs [15] and reducing salt intake by 30% was one of nine global targets endorsed by all WHO Member States [2]. Worldwide, much more sodium than necessary is consumed. The current WHO recommendations are not to exceed 2.3 g per day for adults (5 g of salt per day) while in Europe the overall sodium intake varies between 2.7 and 7.1 g per day (from 7 to 18 g of salt per day) [16]. In Western countries, only 10–12% of sodium intake comes from natural foods, a similar percentage comes from discretionary use at the table, while about 75% comes from processed foods, such as cheeses, cold cuts, and ready-to-eat foods [17]. Sodium reduction is challenging because salt is a multifunctional ingredient in food that is added not only for sensory reasons but also for technological purposes and for the regulation of microbial growth in foods.

### 3.2. Hidden Sources of Salt

There are foods that can heavily impact on the daily intake of salt due to their high frequency of consumption. This is the case with bread and bakery products which are significant contributors to daily salt intake especially in many European countries and in the USA, where daily salt from these foods ranged from 25 to 40% [18]. Other hidden sources of salt are some products with a sweet taste, such as cookies and breakfast cereals [19]. As for sugars, it is important to read the food labels of the products and to choose the products with the lowest salt content.

### 3.3. Salt Substitution and Taste Enhancement Strategies

One of the biggest obstacles to consuming low-salt foods is the biological and learned preference for salty taste [20]. Newborns seem to be indifferent to salty taste probably because of the postnatal maturation of specific central and/or peripheral mechanisms underlying salt taste perception [20]. However, starting from 4–6 months after birth, infants appear to have a preferential response to salty water with respect to plain water [21] and salted baby cereals over plain baby cereals [22], even without a prior exposure to salty taste, suggesting an unlearned biological preferential response to salty taste. During childhood, the increase in preference for salt-rich foods coincides with the higher exposure to salty foods, and for that reason it is thought to be mostly a learned response [20].

Salt content has been reported to affect food preferences among children [23] and the preference for salty taste remains in adulthood [24]. Therefore, lowering the sodium content in food can lead to a significant loss of the acceptability of the food product by the consumer. Changes in consumers’ food choices towards lower sodium intake occur in the case of the low-sodium products matching the characteristics of the normal-sodium products in terms of salty taste and texture [25]. A possible strategy is to replace sodium chloride (NaCl) with potassium chloride (KCl) and other alternative salts such as calcium chloride (CaCl). The limitation of this strategy is the decline in sensory quality caused by the development of metallic and bitter taste. Indeed, NaCl is also described as an effective bitterness inhibitor [26]. Moreover, a risk of toxicity due to higher potassium intake has been described [27].

Due to the influence of sodium reduction on the palatability of foods, another solution could be the addition of flavor enhancers to low-sodium products such as monosodium glutamate (MSG), yeast extracts, hydrolyzed vegetable proteins nucleotides, and amino acids [28]. MSG, which is responsible for the umami flavor, contains one-third the amount of sodium when compared with table salt (NaCl), enabling to reduce sodium by as much as 40%, with no loss of palatability [29]. However, since 1960s, a controversial syndrome, the so-called “Chinese restaurant syndrome” was described as consequence of the use of MSG to flavor most dishes [30]. However, due to the several methodological flaws, and mixed results of most studies on this topic, there is at present limited evidence for an increased human risk after the intake of MSG [31]. The use of yeast extract and hydrolyzed vegetable proteins has limitations due to their specific meaty flavor that is not appreciated by all individuals [32]. The use of essential amino acid (l-lysine mono hydrochloride) in combination with KCl [33] and a mixture of arginine and aspartate [34] were reported with promising results on saltiness, also masking the bitter aftertaste of potassium chloride.

Another strategy could be the replacement of salt by herbs and spices, such as black pepper, oregano and ginger which are rich in flavor and may help reducing the amount of added salt by improving the organoleptic qualities [19,35]. To date, there are few studies with promising results about their acceptability by consumers and the benefit in reducing salt intake [36,37,38,39]. However, these flavorings substantially alter the taste and aroma of food and may not be appreciated by everyone. Furthermore, the use of high amount of specific spices and herbs might lead to toxicity [40].

### 3.4. Reduction Strategy (Gradual Adaptation)

A gradual and progressive reduction over time of flavors has been proposed as a strategy to reduce salt intake. The goal is to shift consumer preference and taste to lower salt intake. The simple exposure to a lower salty flavor intensity for 8 days induces a shift in the preferred salt level [41]. This strategy has been successfully used to reduce the salt content in bread without affecting consumer acceptability [42]. However, the benefits from this strategy can be observed only after a long period of time since salt reduction should be progressive and slow to avoid the decline in acceptance by consumers. Indeed, a permanent shift in the sensory response to salty taste occurred in 4–12 weeks after the start of a low-sodium diet [38]. Changing the taste preference in adults is probably more complex than educating infants to prefer a less salty taste. For this reason, it has been proposed to pre-conditioning the child with a low-salt diet as early as in the weaning period [35]. This period of the life is crucial for setting the taste preferences [43]. Therefore, offering foods low in sugars and salt could be a useful strategy to set the infant’s threshold for sweet and salty tastes at lower levels later in life [44].

Finally, reducing the salt content in food can result in a bitter taste and texture change since salt enhances the taste of other ingredients by masking bitter flavors [45]. Salt also contributes to the textural and rheological properties of cereal-based products by acting on the development of gluten network and by making the dough less sticky and easy to handle [32]. For these reasons, the drastic reduction in salt content in foods is not always a practicable strategy. At the individual level, the strategies to gradually reduce the dietary amount of salt include all the dietary tips listed in Table 1, such as check out food labels for salt and choose lower-salt alternatives, cut down on salty processed foods and ready meals, limit sauces, use little or no salt in cooking, etc. (see below). All these recommendations can be applied gradually over time to promote the adaptation of saltiness perception.

### 3.5. Spatial Distribution

A strategy involving the reduction and the enhancement in taste intensity through non-homogeneous distribution of taste stimuli has been studied. This strategy showed the potential to avoid undesirable changes in sensory properties and in consumer preference [28,46,47,48,49]. The modulation of the spatial distribution of tastants in food matrices is based on the contrast between areas with high and low concentration or the presence of a high concentration of tastants on the surface of the food, and it seems to determine an improvement in taste perception [50]. A greater degree in the heterogeneity of spatial aroma distribution was reported to increase perceived intensity, duration of oral processing, and the saliva content in the bolus [51]. The differences in the concentration of tastants within a food could lead to a discontinuous stimulation of the taste receptors; the partial recovery from taste receptor adaptation and the serial phasic responses have been suggested as the potential mechanisms underlying taste enhancement [32]. The short period during which an uneven distribution of a tastant remains in the mouth, before the formation of a homogeneous bolus, seems to be sufficient to enhance the sweetness and the saltiness perception of the food [51].

A potential strategy for salt reduction is based on repositioning of salt in food matrices. The increase heterogeneity distribution of tastes in various food products, such as sausages and model gels, significantly increased the intensity of taste [52]. The use of inhomogeneous distribution of NaCl in baked products, especially bread, has been widely studied [28]. Breads with an inhomogeneous salt distribution were perceived as saltier than regular bread [28,47,53,54]. Encapsulated salts can be used to create an inhomogeneous sodium distribution in bread. It was observed that the sensory contrast induced by encapsulated salt was able to enhance saltiness and allow for an up to 50% salt reduction [49]. The same author observed that the degree of saltiness enhancement depends on the size of the salt encapsulates: large encapsulated-salt particles lead to a greater sensory contrast than small ones [49]. Thus, encapsulated salts in bread products can be used in the baking process on an industrial scale without modification of the production process in order to reduce salt content while maintaining saltiness and liking of the bread. The effects of salt distribution on saltiness perception in two models of hot snacks (cream-based and cereal-based) was also investigated, and a significant enhancement of saltiness was observed in samples with a heterogeneous salt distribution for both types of snacks [55]. These examples provide evidence that the reduction strategy based on the inhomogeneity distribution could be applied in commercial food products. Furthermore, this strategy could be applied to household food preparation. For example, it might be useful to salt food after cooking, just before the consumption, so that the NaCl crystals remain on the surface.

### 3.6. Cross-Modal Interactions and Food/Flavor Pairing

Liking and food choice are dependent on different factors: the food properties (e.g., appearance, color, texture, aroma, etc.), the consumer (e.g., genetic characteristics, previous experiences, etc.) and the context of consumption (e.g., being with friends, in a restaurant, at home, etc.) [56]. Innate preferences for tastes and favors have been reported by several authors, such as a positive preference for sweet and umami substances, and a negative attitude towards bitter and sour tastes [57,58]. However, learning through experience of different dietary habits (context, frequency, and intensity of different flavors’ exposition, etc.) might have an impact and help modifying the innate preferences; chili, for example, is not harmful but stimulates an innate sensory “warning” system through irritation, which can be enjoyed by those who appreciate the burning but harmless chili sensation [59]. 

Flavor is a cerebral construction resulting from the integration in the brain of chemosensory signals derived from food, such as smell, colors, texture, temperature, and sound [60]. Cross-modal perception is perception that involves interactions between two or more different sensory modalities. In this context, cross-modal odor-taste interactions have been hypothesized for sodium and sugar reduction since olfactory cues might enhance the perception of the salty and sweet taste. Examples of the cross-modal integration between taste and smell have been reported such as the addition of certain odorants to a particular food which can specifically modulate the intensity of a particular taste [50].

The use of aromas could be an efficient strategy to compensate for the reduction of salt content in food products by enhancing salty taste, a phenomenon also known as odor-induced saltiness enhancement (OISE). Several salt-associated odors/aromas have been observed to induce saltiness enhancement, such as cheese [61], soy sauce [62] sardine, ham, and bacon [63]. On the contrary, not congruent odors could induce saltiness reduction, for example carrot aroma showed a decrease of saltiness perception in a low-salt content solution [63]. Therefore, the correct aroma could be used to enhance salty taste in foods containing a small amount of sodium chloride and may compensate for up to a 20% decrease in the food salt content [50]. The intensification of the salty taste with aromas seems to work at low salt concentrations while if the salt concentration is already high, aromas have a weaker effect [64].

In addition, saltiness perception has been demonstrated to be enhanced by tactile cues, indeed improved saltiness impressions have been reported for potato chips with a texture-enhanced product package [65]. The strategy of combining different visual/texture/aroma strategies to favor choice, savory perception, and promote adherence to a weight-loss diet menu was assessed, and an “improved” meal, with the same ingredients and caloric content, but modified appearance, aroma and texture by spices addition or different culinary techniques, gave to increased liking and emotional consumers’ wellbeing than the control meal [66].

### 3.7. Dietary Tips for Salt Reduction 

The dietary tips to reduce salt intake recommended by different national guidelines and the WHO are reported in Table 1. Many tips are common to the different guidelines. Recommendations for reducing salt at the supermarket include choosing fresh products, reducing ready-to-eat products, and checking labels. Furthermore, no salt should be added in the preparation of meals and the use of sodium-rich sauces should be avoided. A few guidelines recommend using spices and herbs [19,67,68]. 

Monitoring salt intake is important both individually, to increase awareness and adherence to recommendations, and at the population level, to evaluate the need and/or effectiveness of large-scale salt reduction programs [69] (Figure 1). Among the several methods, reliable assessments are cumbersome, and simpler methods are less reliable [70]. Measurement of 24 h urine sodium excretion is considered the gold standard, but it is burdensome and expensive, while urinary spot samples can be more easily used [71]. Individual self-monitoring has been performed by nutritional surveys, focused questionnaires, electronic devices, and mobile health (mHealth) technologies. Self-monitoring devices measuring urinary salt excretion allow to estimate 24 h urinary salt excretion from overnight urine samples, morning urine or urinary sodium-to-potassium ratio on spot urine [70,72]. A recent systematic review reported that mHealth interventions had a positive effect on salt reduction in 64% of the 11 analyzed studies [73]. The most common forms of mHealth intervention were short message service (SMS), educational pamphlets, interactive mobile phone applications (i.e., to scan packaged foods/beverages for nutritional information, to monitor diet by daily consumption reporting), or game device to improve disease knowledge, self-management, and behavior [73].

## 4. How to Reduce the Dietary Intakes of Sugar

### 4.1. Dietary Sources of Sugar Intake

A high dietary intake of simple sugars was associated with an increased risk of overweight/obesity [76,77], diabetes mellitus [78], cardio-metabolic risk factors and mortality [79,80], and dental caries [81]. The correlation between sugar intake and arterial blood hypertension is much less well-defined, although there are few studies that have documented this association [80,82,83].

In 2020, the World Health Organization (WHO) published an updated draft guideline confirming the recommendation of a daily sugar intake less than 10% of total energy intake and suggesting additional benefits with a <5% amount per day (corresponding to around 25 g for an adult of normal body mass index (BMI)) [84]. Indeed, worldwide mean sugar intake is much higher than recommended, ranging from 13.5% to 24.6% in adults and from 20.0% to 38.4% in children [85].

It is not known when sugar became the world’s principal sweetener, but the widespread use of sugar has been related to the industrialization and proliferation of processed foods and beverages with added sugar and sweeteners has increased during the last decades [86]. In European countries, major sources of sugars are sweet products (e.g., chocolates, cakes, biscuits and jam) followed by fruits, beverages and dairy products [87]. Sugar-sweetened beverages provide the so-called empty calories, that confer a poor fullness or satiety feeling compared with solid food [79]. The consumption of sugar-sweetened beverages has risen worldwide [80], and several studies showed that the consumption of sugar-sweetened beverages is an important contributor to obesity in both children and adults [88].

### 4.2. Hidden Sources of Sugars

In addition to the previously cited sugar sources, there are several other unsuspected foods that are a source of sugar, which can be discovered by reading the labels. Hidden sugar is found mainly in industrial products since sugar is important either for food preservation or for conferring an adequate food texture [89]. Some examples are soups and sauces (not homemade), canned vegetables, industrial breads, pizza, ready meals, salad dressings and processed meat [90]. Hidden sources provide overall small amount of sugar, but if consumed in large quantities could contribute significantly to the daily sugar intake.

Strategies to avoid hidden sugar include consuming fresh and homemade products, and carefully reading the labels. The list of ingredients may include the terms sugar or sucrose, glucose, dextrose, glucose syrup, high fructose corn syrup, dextrin, corn sweeteners, and malt. Therefore, instructing patients to correctly read labels to avoid high-sugar products is the first step.

### 4.3. Sugars Substitution Strategy

The preference for sweet is innate and express even before birth [91]. From an evolutionary point of view, the inborn preference for sweet taste would promote the acceptance and consumption of breastmilk [20] and, during childhood, may reflect the nutritional need for attracting towards energy-dense foods to maximize the growth [92]. In addition to the innate preference, early and prolonged exposure to sugar foods and beverages leads to an increased preference for sweet taste [93].

The use of alternative sweeteners is a frequently used approach to reduce sugar intake. A wide range of compounds has been used to replace or substitute sugars. Sugar replacement in solid foods requires the combined use of both alternative sweeteners and bulking agents like inulin, polydextrose or maltodextrin [89]. Sweeteners can be divided into two main categories: non-caloric high-intensity sweeteners and nutritive sweeteners. 

High-intensity sweeteners include aspartame, acesulfame-k, neotame, saccharin, sucralose, advantame, and steviol glycosides (derived from stevia leaves). This group of sweeteners are 200 to 13,000 times sweeter than sugar and are often referred as non-nutritive sweeteners [94]. Potential safety concerns have been raised regarding the possible relationship between the consumption of non-caloric high-intensity sweeteners and the development of adverse effects, including carcinogenicity [95]. Moreover, several studies have found a correlation between artificial sweetener use and weight gain [96], and risk of type 2 diabetes [97]. Furthermore, sweetness decoupled from caloric content offers partial, but incomplete activation of the food reward pathways, which may further fuel food seeking behavior, encouraging sweet cravings and sugar dependence from other foods [98]. Moreover, sweeteners could cause glucose intolerance through induction of compositional and functional alterations to the gut microbiota [99,100]. In animal studies, saccharin and sucralose have been led to a shift of the gut microbiota with a decrease in the total number of anaerobic and aerobic bacteria, *Bifidobacterium*, *Lactobacilli*, *Bacteroides*, *Clostridium* and *Akkermansia muciniphila* [100]. Sweeteners-induced dysbiosis could be the cause of altered metabolic pathways linked to glucose intolerance. However, further studies, especially human clinical trials, are needed to elucidate the effects of sweeteners on the human gut microbiota.

Nutritive sweeteners include sugar alcohols, such as sorbitol, xylitol, maltitol, mannitol, and erythritol. Sugar alcohols are slightly lower in calories than table sugar, and their sweetness varies from 25% to 100% of that of sucrose. Sugar alcohols are not fermentable by oral bacteria, do not promote dental caries, and are primarily used to sweeten “sugar-free” candies, chewing gums and cookies. These compounds when used in high quantities can have a laxative effect, but no other adverse health effect has been reported, differently from the high-intensity artificial sweeteners; therefore, an acceptable daily intake (ADI) has not been determined [101]. Furthermore, moderate doses of polyols, including isomalt and maltitol, could induce an increase in the relative abundance of *Bifidobacterium* and may act as a prebiotic in healthy subjects [100]. 

Sweeteners are regulated as food additives by the European Food Safety Authority (EFSA) in Europe and by the Food and Drug Administration (FDA) in USA. A few legislation discrepancies between FDA and EFSA exist. The FDA has not approved the use of cyclamate as a sweetener [102]. Moreover, the ADI values are generally higher for the FDA than for the EFSA (Table 2).

### 4.4. Spatial Distribution Strategy

To date, non-homogeneous distribution of taste stimuli is used mainly for salt as described before, while for sugar reduction, this approach has been adopted in gel matrices only. A different sugar concentration between layers in models of gels was able to enhance early sweetness intensity compared to gels with homogeneous sugar distribution [46]. Moreover, models of gels with large concentration gradients between layers were perceived sweeter than homogeneous gels, while no differences in sweetness were observed between gels with small concentration gradients [48]. The spatial distribution strategy allowed a 20% sucrose reduction in gelled products without compromising sweetness intensity [104]. Furthermore, consumers equally or even more preferred food with inhomogeneous distributions of tastants than products with homogeneous distributions with the same tastants concentration; furthermore, inhomogeneity is not perceived by consumers [104]. Moreover, an important role is played by the oral breakdown of food that contributes to the release of tastants from food matrix and allows the contact of tastants with taste receptors. It was demonstrated that gels that break into a large number of small fragments during chewing had the highest sweetness intensity suggesting that the breakdown behavior of the gel matrix during oral processing affects the perception of sweetness of layered gels [105].

### 4.5. Cross-Modal Interactions and Food/Flavor Pairing

The characteristic taste inclinations for sweet substances might reflect a biological drive towards caloric foods, maybe because these foods are concentrated sources of energy with a rewarding post-ingestion effect [106], but this innate preference can be modified by education and cultural or culinary habits.

The human brain learns to bind combinations of olfactory and gustatory stimuli that have been tasted together in the foods that the person has already experienced [107]. Olfactory stimuli that have regularly been paired with sweet, bitter, salty, or sour-tasting foods could enhance the associated taste quality, even when they are presented at a sub-threshold level [108]. Therefore, using natural aromas in the food reformulation might increase the sweet perception of the product, but the olfactory stimuli need to have been previously paired with the sweet taste (culture/habits dependent). Flavor and expectations, and therefore acceptance, depend on previous experiences and on their memorization. In addition to the food properties, it is important to take into consideration consumers’ memory of taste for determining how much it is possible to reduce the content of some ingredients such as fat, sugar, or salt [109].

Therefore, a novel strategy to reduce sugars content in food is based on this multisensory integration. Consequently, pairing foods with specific aroma could help reduce the amount of sugar. Specific odors such as strawberry [110], vanilla [111,112,113,114,115,116,117,118,119], caramel, maracuja, and lychee [120] have been observed to enhance sweet taste intensity. A weakening of the sweet taste intensity was also observed with angelica oil and damascone, a flavor used in wine [120]. However, sweetness enhancement by odors allowed only a small reduction of sugar intake compared to non-nutritive sweeteners [121]. To obtain a better result, the combination of the two strategies (the use of non-nutritive sweeteners and aroma together) has been proposed [122]. In addition, in the same manner that combining odor and taste sensory modalities, using visual or textural cues could help enhancing sweetness. Indeed, the texture of the container (e.g., cup) has been reported to enhance the sweetness perception of iced tea through the cup’s surface [123]. Additionally, Velasco et al. [124,125] showed the correspondence of different visual cues (colors and shapes) with different tastes, being, for example, red colors and curvy shapes related with sweetness. Including different sensory modalities which enhance sweetness can be used by industry, combining not only the intrinsic properties of the product, but also packaging and/or cutlery properties as product reformulation strategies. 

### 4.6. Dietary Tips for Sugars Reduction

Worldwide, nutritional guidelines and government website contain recommendations for limiting salt and sugars in diets. The recommendations for sugar reduction are summarized in the table below (Table 3). 

Several recommendations are shared by different national guidelines [19,67,68,74,75]. Almost all documents recommend to checking food labels in order to choose low-sugar foods and avoiding sugar beverages [19,67,74,75]. A few tips are specific to certain guidelines because they depend on typical eating habits (for example the use of cinnamon) [75]. Other recommendations differ among guidelines, e.g., the Italian guidelines do not recommend the use of artificial sweeteners [19] while the UK guidelines suggest the use of low-calorie sweetener [67]. 

Self-monitoring of sugar intake by carbohydrate counting is taught and recommended to patients with diabetes mellitus who may have feedback on their nutritional habits through postprandial blood glucose and glycated hemoglobin measurements [126]. In the general population, sugar intake is monitored more roughly using food frequency questionnaires, food logs, and 24 h reminders [127], which are the tools used for national surveys to assess the efficacy of public health interventions [87,128,129]. Urinary fructose and sucrose, measured on 24 h urine collection, have been proposed as more reliable predictive biomarkers of sugar intake at both the population level [130] and for research purpose [127].

## 5. Future Perspectives

Reducing the sugar or salt intake among the population will be achieved after implementing different strategies promoted by different government policies (e.g., United Kingdom [131,132,133]). The European Union also favors the development of research and technology projects related to food improvement, looking for the development and application of updated scientific knowledge [134]. Most of the actions include reformulation, targeted taxation, and interpretative front-of-pack labeling. Processed food reformulation has been underway for many years, sometimes driven by the food and beverage manufacturers in response to changing consumer preferences, and, sometimes, as a result of government policies. The aforementioned collaborative plans push the reformulation to the next years, leaving industry the task of designing healthier products, and assuming the risk of losing consumers if the reformulation does not succeed. However, besides promoting products reformulation, it is essential the consumers’ involvement through education plans favoring healthier food choices. In particular, adding better health education efforts showing the health risks and benefits of dietary salt and sugar in primary and secondary education should be tested to see if this will help in lowering their consumption later in life. Individual behavior change needs a supportive environment, including communities, schools, and workplaces, to make healthy food choices [135]. It is therefore time to consider global strategies, based on lifestyle programs where preferences, nutritional and cooking aspects are taken into account to enable a health behavior change in individuals at increased cardiometabolic risk. Other than individual reduction strategies, and practical recommendations for lowering salt/sugar intake, programs based on culinary medicine, could be a good starting point to impact on the health of patients and community through a multidisciplinary approach [136,137]. Mindfulness-based eating interventions may be also worth considering as a strategy to modulate eating habits [138] and may have potential to strengthen the effects of cardiovascular health promotion programs [139,140].

## 6. Conclusions

Due to their impact on the cardiometabolic risk, avoiding the consumption of excessive salt and added sugars should be clearly defined in dietary recommendations. Along this line, both individuals at increased cardiometabolic risk and the whole population should be aware that most of the salt and sugar introduced with the diet does not come from the amount added while cooking or dressing, but mainly from processed foods. In this regard, the best behavior would be to substitute these processed foods with natural products cooked and dressed at home. However, the profound changes that have occurred in family organization, with nearly all the components being out of home for most of the day (at school or work), together with the pounding advertising of ready-to-eat foods on newspaper, television, and internet, do not facilitate the change of dietary habits. The strategies to reduce both sugar and salt consumption herein presented could be a useful tool to improve dietary habits. Indeed, the issue is addressed from two different points of view: (1) increasing patient awareness (substitution of salt/sugar with other dressings); and (2) providing advice to the food industry in order to take care of visual, texture and aroma strategies that will facilitate patient adherence to a healthy diet. Particularly relevant, in this regard, is the observation that combining food interventions (substitution of salt/sugar with other dressings) with visual, texture and aroma strategies results in improved adherence to a healthy diet. Last, but not least, since the existing national guidelines do not always share the approach of managing the amount of dietary sugar and salt (see above), a comprehensive approach aimed at reaching a consensus on this issue would be welcome. 

## Figures and Tables

**Table 1:** Tips for salt reduction according to different national guidelines and the WHO.

**At the Supermarket**	Cut down on salty processed foods and ready meals [67] Limit sauces, mixes, and “instant” products, including flavored rice and ready-made pasta [68] Check out food labels for salt and go for lower salt choices; compare salt levels among similar products and try to choose those lower in salt [16,67,74] Go for reduced-salt unsmoked back bacon; cured meats and fish can be high in salt, so try to eat these less often [67] When buying prepared meals, look for those with less than 600 milligrams (mg) of sodium per meal [68] Buy fresh, frozen, or canned vegetables with no salt or sauce added [67,68] Switch salty snacks (crisps, biscuits) for fruit, veggie sticks or non-added salt alternatives [67,68] Try unsalted snacks, such as unsalted popcorn or unsalted nuts [67] When possible, purchase fresh poultry, fish, pork, and lean meat, rather than cured, salted, smoked, and other processed meats. For fresh items, check to see whether saline or salt solution has been added—if so, choose another brand [68] Ask your grocer if they have a low sodium shopping list available [68]
**Cooking**	Use little or no salt in cooking [16,19,67,68,74,75] Limit the use of soy sauce, products with glutamate and other sauces rich in salt [19] Use alternatives such as garlic, citrus juice, salt-free seasonings, or spices [19,67,68] Lemon juice and vinegar enhance the flavor of foods [19] Use black pepper as seasoning instead of salt. Try it on pasta, scrambled egg, pizza, fish and soup [67] Tomato-based sauces for pasta are often lower in salt than cheesy sauces or those containing olives, bacon, or ham [67] Try baking or roasting vegetables such as red peppers, tomatoes, zucchini, fennel, parsnips and squash to bring out their flavor [67]
**At Table**	Leave the saltshaker off the table [67] Be wary of gourmet salts and salt substitutes claiming to be better for your health than table salt—these product ranges are still likely to add some form of salt to your diet [19,67] Instead of using stock cubes or gravy granules, why not make your own from scratch or use low salt versions [67] Limit the use of alternative seasonings containing sodium (bouillon cubes, ketchup, soy sauce, mustard, etc.) [19] Reduce your portion size—less food means less sodium [68] Consume only occasionally processed foods that are rich in salt (salty snacks, crisps in bag, table olives, some processed meat and cheese) [19]
**At Restaurant or Eating Out**	Removing saltshakers and soy sauce from tables in restaurants [16] If you are eating in a restaurant or cafe, or ordering a takeaway, you can still eat less salt by making smart choices of lower-salt foods [67]: Pizza: choose vegetable or chicken toppings instead of pepperoni, bacon, or extra cheese Pasta dishes: choose one with a tomato sauce with vegetables or chicken, rather than bacon, cheese, or sausage Burgers: avoid toppings that can be high in salt, such as bacon, cheese, and barbecue sauce, and opt for salad instead Chinese or Indian meal: go for plain rice. It is lower in salt than pilau or egg fried rice Sandwiches: instead of ham or cheddar cheese, go for fillings such as chicken, egg, mozzarella, or vegetables like avocado or roasted peppers. Try having salad and reduced-fat mayonnaise instead of pickle or mustard, which are usually higher in salt Breakfast: instead of a full English breakfast, go for a poached egg on toast with mushrooms and grilled tomatoes. If you do have meat, have either bacon or a sausage, but not both Salad: ask for dressings or sauces on the side, so you only have as much as you need Some dressings and sauces can be high in salt and fat

**Table 2:** Sweetener types and characteristics [102,103].

Sweetener	Sweetness Compared to Table Sugar	kcal/g	ADI (mg/kg Body Weight/day)	Comment
**Non-nutritive high-intensity sweeteners**				
Acesulfame K	×200	0	15 (FDA) 9 (EFSA)	Can be used for cooking and baking; bitter taste
Aspartame	×200	4	50 (FDA) 40 (EFSA)	It is not heat-stable and loses its sweetness when heated, so usually it is not used in baked goods. It is a source of phenylalanine. People with phenylketonuria should control their intake of phenylalanine from all sources, including aspartame
Saccharin	×200–700	0	15 (FDA) 5 (EFSA)	It is the first to be discovered (1879). Suitable for cooking or table use
Neotame	×7000–10,000	0	0.3 (FDA) 0–2 (EFSA)	Derivative of aspartame. It is heat-stable
Cyclamate	×30–50	0	Banned by FDA 7 (EFSA)	To improve palatability cyclamate is often blended with saccharin
Sucralose	×600	0	5 (FDA) 4 (EFSA)	It is heat stable
Advantame	×20,000	0	32.8 (FDA) 5 (EFSA)	FDA-approved in 2014. It is heat-stable
Steviol glycosides	×200–400	0	4 (EFSA) * 4 (FDA) *	It can be used for cooking and baking
**Sugar alcohols**				
Erythritol	0.7	0.2	NA Maximum non-laxative dose 0.66–1 g/kg body weight	Stable at high temperatures. Found naturally in fruits, vegetables, mushrooms, and fermented foods (wine, soy sauce). Used as bulk sweetener in several low-calorie foods
Sorbitol	0.6	2.5	NA Maximum non-laxative dose 0.17–0.24 g/kg body weight	Stable at high temperatures. It is commonly used in dietetic foods including ice cream and diet drinks, sugar-free chewing gum, mints, and cough syrups
Isomalt	0.45–0.65	2	NA Maximum non-laxative dose 0.3 g/kg body weight	Stable at high temperatures. Used in hard candies, toffee, chewing gum, chocolate, and cough drops
Maltitol	0.75	2.7	NA Maximum non-laxative dose 0.3 g/kg body weight	Stable at high temperatures. Obtained from starch by hydrogenating maltose.Used in hard candies, chewing gum, chocolates, baked goods, and ice cream
Xylitol	1	2.5	NA Maximum non-laxative dose 0.3–0.42 g/kg body weight	Stable at high temperatures. It is commonly used in chewing-gum

ADI: acceptable daily intake. FDA: Food and Drug Administration. EFSA: European Food Safety Authority * ADI established by the Joint FAO/WHO Expert Committee on Food Additives (JECFA).

**Table 3:** Tips for sugar reduction according to different national guidelines.

**At the Supermarket**	Choose unsweetened foods and drinks [75] Among baked foods, choose those with the lower sugar and fat content [19] Check nutrition labels to help you pick the foods with less sugar, and choose foods with little to no added sugars [58,59] or go for the low-sugar version [74] Looking at food labels can really help you to choose foods and drinks that are lower in sugar [67] Choosing beverages with no added sugars, such as water, in place of sugar-sweetened beverages, reducing portions of sugar-sweetened beverages, drinking these beverages less often, and selecting beverages low in added sugars. When juices are consumed, they should be 100% juice, without added sugars. Additionally, when selecting canned fruit, choose options that are lowest in added sugars [68] Consuming unsweetened foods and drinks is especially important for children. Foods consumed at an early age can influence their taste preferences and lifelong eating habits [75] Swap biscuits for oatcakes, oat biscuits, or unsalted rice cakes, which also provide fiber [67] Choose wholegrain breakfast cereals, but not those coated with sugar or honey [67,74]
**Cooking**	Reduce the amount of sugar you add to drinks [19,75] Avoid/limit the consumption of sugar substitutes [19] Try halving the sugar you use in your recipes. It works for most things except jam, meringues, and ice-cream [74] Choose tins of fruit in juice or water rather than syrup [67,74] Sweetening foods naturally by using fruits; try this with yogurt, oatmeal, baked goods [75] Adding flavor by using ingredients such as nutmeg, cinnamon, vanilla extract [75]
**At Table**	Avoid sugar-sweetened beverages [19] You could try flavoring water with a slice of lemon, lime, or a splash of fruit juice. But watch out for the sugar content in flavored water drinks: a 500 mL glass of some brands contains 15 g of sugar—nearly 4 teaspoons of sugar [67] Use a small amount of spreadable sweet cream, jam, and honey [19] Rather than spreading jam, marmalade, or honey on your toast, try a scrape of low-fat spread or sliced banana instead [67,74] Swap cakes, biscuits, and desserts for a piece of fruit [74,75] If you take sugar in hot drinks or add sugar to your breakfast cereal, gradually reduce the amount until you can cut it out altogether or try using a low-calorie sweetener [67] Try some new flavors with herbal teas, or make your own with hot water and a slice of lemon or ginger [67] Limit or decrease portion size of grain-based and dairy desserts and sweet snacks and choose unsweetened or no-sugar-added versions of canned fruit, fruit sauces (e.g., applesauce), and yogurt [68] Dried fruit, such as raisins, dates, and apricots, is high in sugar and can be bad for your dental health because it sticks to your teeth [67]

## Data Availability

Not applicable.
